# Quantifying the impact of environment factors on the risk of medical responders’ stress‐related absenteeism

**DOI:** 10.1111/risa.13909

**Published:** 2022-03-14

**Authors:** Mario P. Brito, Zhiyin Chen, James Wise, Simon Mortimore

**Affiliations:** ^1^ Department of Decision Analytics and Risk University of Southampton, Centre for Risk Research Southampton UK; ^2^ South Central Ambulance Service, NHS Foundation Trust, Southern House Otterbourne Sparrowgrove UK

## Abstract

Medical emergency response staff are exposed to incidents which may involve high‐acuity patients or some intractable or traumatic situations. Previous studies on emergency response staff stress‐related absence have focused on perceived factors and their impacts on absence leave. To date, analytical models on absenteeism risk prediction use past absenteeism to predict risk of future absenteeism. We show that these approaches ignore environment data, such as stress factors. The increased use of digital systems in emergency services allows us to gather data that were not available in the past and to apply a data‐driven approach to quantify the effect of environment variables on the risk of stress‐related absenteeism.

We propose a two‐stage data‐driven framework to identify the variables of importance and to quantify their impact on medical staff stress‐related risk of absenteeism. *First*, machine learning techniques are applied to identify the importance of different stressors on staff stress‐related risk of absenteeism. Second, the Cox proportional‐hazards model is applied to estimate the relative risk of each stressor. Four significant stressors are identified, these are the average night shift, past stress leave, the squared term of death confirmed by the Emergency Services and completion of the safeguarding form. We discuss counterintuitive results and implications to policy.

## 1 INTRODUCTION

Stress is a very common challenge in all professions nowadays, particularly in the field of emergency medical response services. Paramedics or emergency staff are frequently exposed to incidents that are detrimental to mental health (Firth & Britton, 1989). Stress‐related absenteeism can cause productivity loss and poses a financial risk to the medical response providers. The United Kingdom National Health System (NHS) provider loses £2.4 billion per year due to health‐related absenteeism (Questback, 2021). It is important to understand medical responders’ absenteeism risk because, in addition to the financial constraint, there is also the risk of medical errors because of work overload (Kazemi et al., 2017).

Several studies have demonstrated that emergency medical services staff, including ambulance crew or clinicians in emergency departments, constitute a large group whose members are exposed to inconceivable stress, which has long been considered a key cause of sickness absence in many fields (Luz & Green, 1997). Sedigheh et al. (2013) showed that the majority of paramedics and hospital emergency personnel have experienced post‐traumatic stress disorder (PTSD) with the incidence rate up to 94%. Sterud et al. (2006) study revealed that, in Sweden, the ambulance staff were more prone to suffer from PTSD than the general population was. The incidence rates of these two groups were 21.5% and 2.6%, respectively (Sterud et al., 2006).

With a vast number of studies on PTSD in medical responders and with the large number of models for absenteeism prediction it is surprising that, to date, there is no published methodology for managing the risk of stress‐related absenteeism of medical emergency response staff due to stress factors based on hard data. Instead, absenteeism risk is managed by factors identified in workshops and surveys. The limitation with current practice is that there is no hard evidence that some of these stressors cause stress‐related absenteeism. With environment data stored in multiple digital systems it is now possible to explore which environment factors may have stress‐related absenteeism. This can subsequently inform the implementation of risk response practices. Absenteeism prediction models that predict absenteeism based on *past* absenteeism do not capture stressors that may cause absenteeism in medical emergency responders in today's environment. To develop the risk management practice for managing medical responders’ absenteeism we must improve absenteeism risk prediction models. In this paper we propose a process for identifying and quantifying causes of medical responders’ risk of absenteeism based on hard data acquisition of environment factors stored in a diverse set of digital systems. The framework is underpinned by a more detailed risk of absenteeism model.

Failure of labor management is one of the key organizational factors that underpins medical errors (Inoue & Koizumi, 2004). If the risk of different causes of stress‐related absenteeism can be predicted, this will benefit both the employees and the employers. The emergency response organization can duly provide supportive care or professional psychological intervention to their employees, helping them to deal with their mental health issues and prevent negative outcomes.

Recent research has focused on the prediction of risk of adverse events in medical settings. Kazemi et al. (2017) proposed a methodology that combines Systems dynamics (SD) simulation models and Bayesian belief networks (BBNs) to estimate the dynamic impact of adverse events on staffing, length of stay, and investments in safety. Two types of adverse event were considered—*pressure ulcers* and *vascular‐catheter associated*. The models were validated based on expert opinion. However, although their research addresses several risk factors, it does not address the issue of emergency medical staff stress‐related absenteeism.

Research on data‐driven risk of absenteeism prediction has focused on the application of two types of methods—*regression* methods (Roelen et al., 2011) and *classification* methods (de Oliveira et al., 2019). The key limitation with previous studies is that they do not take account of different stressors. Also, previous research has not attempted to predict stress‐related absenteeism for medical emergency response staff.

We propose the use of the Random forest (RF) model to identify key stressors in the risk of absenteeism and the use of Cox regression (Cox, 1972) for absenteeism risk calculation. RF is adopted in operations research for dimensionality reduction (Antoniadis et al., 2021). Antoniadis et al. (2021) reviews articles that use RF for performing model sensitivity analysis to inform dimensionality reduction. We follow a similar approach to address the limitations of the Cox proportional‐hazard model.

The paper is organized as follows. In section 1 we present the research literature in stress‐related leave prediction and the limitations with the current approaches. In section 2 we present the proposed methodology while the data collected for this research are reported in section 3. The outcomes and performances of all the models are critically compared and carefully expounded in section 4. All the significant findings, strengths, and limitations, and advice for future work are discussed in section 5. Finally, we present the conclusions in section 6.

### 1.1 Regression models for risk of absenteeism

Most articles that attempt to estimate risk of absenteeism adopt the risk definition presented in Kaplan and Garrick (1981) where risk is defined by the triplet consisting of Scenario (Xi), Likelihood (Li), and Consequence (Ci). The scenario is past absenteeism; for example, the cumulative absenteeism recorded in the previous year. The Likelihood (Li) is the probability of observing an absenteeism in the current year given the past absenteeism. The consequence (Ci) is the absenteeism taken in the current year. Roelen and colleagues (2012) found that when using self‐rated health, prior sickness absence episodes and age as predictors to forecast sickness absence episodes, the overall performance of logistic regression evaluated by the Nagelkerke's pseudo *R* ^2^ was 31.8% (Roelen et al., 2012). The area under receiver operating characteristics (AUROC), an index used to measure the model's predictive ability, was 0.831 with 95% confidence intervals between 0.784 and 0.877 (Roelen et al., 2012). Research has indicated that prior sickness absence could serve as a strong predictor of further sickness absence because it could explain up to 30% of the variance in the observed absenteeism (Reis et al., 2011). It was also reported that there was a negative relationship between self‐rated health and high sickness absence episodes (Roelen et al., 2012).

Roelen et al. (2011) used ordinal regression to explore if past sickness absenteeism can predict future sickness absenteeism. The authors measured past sickness absenteeism in terms of number of days of absenteeism and frequency of absenteeism. The authors concluded that the number of days of absence in a given year were positively associated with the number of absences in the previous year. They have also concluded that the frequency of sickness absence in the preceding 2 years made a significant contribution to the prediction of sickness absence episodes in the current year (Roelen et al., 2011).

Poisson regression is another model utilized to predict sickness absence. Research showed that decision authority, predictability, and meaning of work (one of the psychosocial work environment factors) could be used to predict sickness absence in a statistically significant way. A one unit increase in the standard deviation of decision authority could lead to a reduction of number of sickness absence incidents by 13% (Christensen et al., 2007). However, there are some limitations when using Poisson regression; Christensen et al. (2007) observed that Poisson regression underestimated the true effect size of the data by approximately 10% by ignoring two important aspects. *First*, it failed to consider that a person may have multiple sickness absences and the potential correlation among these absences. *Second*, the risk of sickness absence could vary when a person returned to work after a sickness absence.

Nieuwenhuijsen et al. (2006) used Cox regression to predict the risk of absenteeism. Their data were derived from a cohort of 188 individuals, of which 102 were teachers on sick leave with common mental health disorders. The authors applied Cox regression to predict the risk of absenteeism consequent on underlying mental health disorders. For this study, the scenarios consisted of four significant factors: (i) aged over 50; (ii) expectation of long‐term sickness absence; (iii) higher educational level, and (iv) diagnosis of depression or anxiety disorder. The Ci in this study was the duration of the absenteeism and the triplet definition of risk was adopted (Kaplan & Garrick, 1981). The Li of absenteeism given each of the four factors was quantified. The AUROC of this model was approximately 0.7, which implied an acceptable predictive ability. In this context, a normal Cox regression model might lose valuable information. In the methodology section, section 2, we propose a solution to this problem.

### 1.2 Classification models for risk of absenteeism prediction

Studies have used prominent tree‐based machine learning methods including decision tree, gradient boosted Tree, RF, and tree ensemble (Wahid et al., 2019) to predict absenteeism time of employees at work. Wahid et al. (2019) concluded that gradient boosted tree had the best predictive ability with accuracy up to 82%. The accuracy of tree ensemble was the lowest, but it was still 79% (Wahid et al., 2019; Zaman et al., 2019). Gayathri (2018) used absenteeism data from the University of California, Irvine (UCI) data repository to create several prediction models based on different machine learning algorithms—namely, naive Bayes and multilayer perceptron (MLP). The accuracy of MLP is 97%, proving more effective than naive Bayes. In addition, the root mean square error of MLP is subtle—only 0.0969 (Gayathri, 2018). Another study by de Oliveira et al. (2019) which focused on absenteeism in call centers also applied several machine learning algorithms and critically compared them. With a population of 13805 employees and 241 features included in the model, the authors employed RF, MLP, support vector machine (SVM), naive Bayes, XGBoost, and long‐short‐term memory (LSTM) to build up the prediction model. The findings demonstrated that XGBoost and RF were the best‐performing algorithms, with an accuracy of 72% and 71%, respectively, while LSTM and SVM were the least accurate, with an accuracy of 53% and 56%, respectively (de Oliveira et al., 2019).

Indeed, ample research investigated the factors that evoke stress in emergency medical services. Such stressors can be, for example, child death and completion of a safeguarding form. The latter is a process used for reporting child welfare concerns. To the best of our knowledge, no published research uses a data‐driven approach to estimate the risk of absenteeism among medical emergency responders taking into consideration these and other factors. In this paper we propose an approach to address this problem.

### 1.3 The present study

In the previous section we showed the limitations of regression models and machine learning classification models for absenteeism risk prediction. The Cox regression is the most suitable method for predicting risk of absenteeism; however, its key limitation is that it is too sensitive to the number of parameters. When we attempted to apply Cox regression, we observed that, for a large number of parameters, it is hard for the model to meet the assumption of proportionality. Methods to test the proportionality assumption, such as the log‐cumulative hazard plot, are easier to operate when there is a small number of factors (Collett, 2003a). Machine learning classification methods such as XG boosted trees and RF have shown to have a high accuracy in absenteeism classification. These methods do not allow probabilistic quantification of the risk of absenteeism as they tend to overestimate or underestimate the probability of occurrence, although they are suitable to identify key stressors.

Therefore, we propose a two‐stage framework for medical emergency response staff risk of absenteeism. In the first stage we apply machine learning classification methods to identify key stressors in the risk of absenteeism. In the second stage, we apply Cox regression to estimate the risk of absenteeism due to different key stressors.

In this paper, the application of Cox regression is different from that seen in previous published survival research. In conventional statistical survival recording, there is only one death per entry, whilst in absenteeism risk prediction a person can take several periods of stress‐related leave and, therefore, there may be several deaths per entry. In past applications of the Cox regression, a person usually has one record, but in this research, it is likely that a person can have more than one record in the dataset. We give a detailed description of the dataset in Section 3, and we also detail the process for flagging up instances of stress‐related leave. This can fully capture the inducement of each incidence of stress‐related leave, thereby avoiding information loss.

## 2 METHOD FOR DEVELOPING A FRAMEWORK FOR EMERGENCY MEDICS’ RISK OF ABSENTEEISM

Instead of determining one specific model to conduct the prediction, we applied a more explorable approach. Both machine learning methods and statistical model were applied to build up the stress‐related leave prediction model and their performances were critically compared. Specifically, the two models were RF and Cox regression. The first originated from machine learning and the second originated in medical statistics.

### 2.1 Random forest

RF machine learning algorithm creates abundant decision trees with various classification rules (Brid, 2018; Ho, 1995). Each decision tree contains nodes, branches, and leaves. *Node* represents a conditional statement associated with features in the model (Brid, 2018). A population is subgrouped by these nodes. *Branch* exhibits the outcomes of whether a subgroup satisfies the conditional statement shown on the node. *Leaf* is the termination of the tree, telling us the class label. By following each branch from root to leaf, it is easy to understand the classification rules (Brid, 2018).

Based on the outcomes of these decision trees, the RF gives a prediction which is the majority vote on these outcomes (Ho, 1998). The advantage of the RF is that it uses different training set samples to train each tree. There is an inherent bootstrap sampling in its algorithm, resulting in an unbiased estimation and avoiding overfitting.

### 2.2 Cox regression

Cox regression, also called the proportional hazard model, is widely used in medical research to investigate how risk factors affect the time of the event of interest (Collett, 2003b); for example, whether taking a new medicine can extend the survival time of a cardiovascular patient. Unlike other models for survival analysis, not only categorical variables but also continuous variables can be included in the Cox regression model. Since Cox regression is a branch of survival analysis, it can consider the event status (whether the event occurs or not) and the time before the event occurs. One advantage of Cox regression is that there is no strict requirement for the data distribution. Cox regression can use data that are not normal‐distributed (Collett, 2003b). Like any other survival modeling technique, the data are censored or not censored. If the concerned event of an individual has not been observed at the end of the observation period, the survival time of this individual is considered as censored.

The function *h*(*t*) represents the hazard of concerned event at time *t*. This is calculated using the following equation:

(1) $$h\left(t\right)=h_{0\;}\left(t\right)\times exp\left(b_{1}x_{1}+b_{2}x_{2\;}+\cdots +b_{n}x_{n}\right)$$

where *h*_0_(*t*) is the baseline hazard function. It indicates an individual's hazard at time *t* when all the covariates included in the model are equal to 0. $x_{1}$ to $x_{n}$ stand for the covariates of the event of interest. $b_{1\;}$to $b_{n}$ measure the effect of the covariates. The baseline hazard function and coefficients of covariates are all estimated by using the method of maximum likelihood (Collett, 2003a).

To apply Cox regression, a crucial assumption must be verified before drawing any conclusions from the model. The assumption is that the hazard ratio of two individuals is constant over time (Cox, 1972). If the assumption is violated, it means that the linear component of the model may fluctuate over time (Collett, 2003c). The Schoenfeld residuals plot helps verify whether this condition is met.

After fitting the Cox regression model, the coefficients of factors denoting the impact and the probability of stress‐related leave can be readily obtained by calculating the hazard function. The survival function of different groups of the sample could also be estimated by plotting Kaplan Meier survival distribution (Kaplan & Meier, 1958). A log rank test was used to see whether the survival functions of different groups were notably different.

### 2.3 Performance of the prediction models

To measure the performance of a prediction model, it is essential to evaluate two aspects—*discrimination* and *calibration* (Alba et al., 2017). Discrimination assesses how well a model can correctly distinguish different categories. If a model estimates the probability of subjects who have the event higher than those who do not have the event, it means that the model's discrimination is good (D'Agostino & Nam, 2003). A model with ideal discrimination can generate two sets of predicted probabilities which are not overlapping. To explain, one set is for the true positive outcomes and the other is for the true negative outcomes (D'Agostino & Nam, 2003). There is no false positive or false negative from the perfect prediction model. In this study, discrimination refers to the ability of the model to identify ambulance crew who take stress‐related leave from those who do not take stress‐related leave.

Sensitivity, specificity, positive predictive value, negative predictive value, accuracy, receiver operating characteristic (ROC) curve, and areas under the ROC curve (AUROC) can reflect the model's discrimination in some way. The ROC curve and the AUROC were regarded as the measures for discrimination in this study. The ROC curve plotted sensitivity versus 1 – specificity for each possible cut‐off where sensitivity indicated the true positive rate on the y axis and 1 – specificity stood for the false positive rate on the *x* axis. The criterion of discrimination measured by the AUROC is shown in Table 1 (Roelen et al., 2012).

**TABLE 1:** Discrimination ability classification by area under receiver operating characteristics (AUROC)

Discrimination ability classification	
0.9 < = AUROC < 1.0	Excellent
0.8 < = AUROC < 0.9	Good
0.7 < = AUROC < 0.8	Fair
0.6 < = AUROC < 0.7	Acceptable
AUROC < 0.6	Fail

However, the AUROC was only applied to evaluate the discrimination ability of the RF. The measurement of Cox regression in this aspect was slightly different since Cox regression allows censorship. Instead, an assessment called concordance index (*C*‐index) is used to solve this problem. The *C*‐index is widely used to reflect the predictive ability of survival analysis (Koziol & Jia, 2009). The value of the *C*‐index denotes the probability that a subject who has experienced the concerned event has a higher risk score than a subject who has not experienced the concerned event. It plays a similar role to that played by the AUROC relating to survival data, with the consideration of, and ranges from, 0.5 to 1. A rough guide for understanding the C‐index is that *C*‐index < 0.5 = fail; *C*‐index 0.5–0.6 = better than random prediction; *C*‐index 0.6–0.7 = fair; *C*‐index 0.7–0.8 = good, and *C*‐index > 0.8 = excellent.

Calibration is a model performance measurement that describes how close the agreement between the observed and predicted outcomes is (Rahman et al., 2017). When the *predicted* probability is close to the *real* probability, the model is regarded as well calibrated (Rahman et al., 2017). Calibration can be reflected by a calibration curve where the *x* axis is the sum of real outcomes while the *y* axis is the sum of the model's estimates. The closer the calibration curve is to the diagonal line, the better the calibration is.

In this study, calibration curve and Hosmer–Lemeshow tests were both used to assess the calibration ability. The Hosmer–Lemeshow test drew a comparison between the predicted probability of taking stress‐related leave and observed probability, with the null hypothesis that predicted probabilities were equal to the observed probabilities (Hosmer & Lemeshow, 2013).

## 3 DATA

The data considered in this study were collected from the South Central Ambulance Service NHS Foundation Trust (SCAS) in the United Kingdom. The focus was on stress that was reported among the ambulance staff and the stressors that the ambulance staff confronted in their daily work. The study population was all the ambulance staff working in the SCAS, which consisted of 1431 individuals.

The variables included in the model were identified based on discussions with one Business Intelligence Analyst at SCAS, one 999 Business Intelligence Analyst, and interviews with the Head of Operations Berkshire West and lead for SCAS trauma risk incident management (TRiM) team. The TRiM team provides support to SCAS staff who have been involved in traumatic incidents. Staff can self‐refer, or their manager can refer them for support. The interviews gave us very valuable insights into what staff find traumatic; this directly influenced the identification of incidents that involved child death, childbirth, and safeguard (such as abuse). Details of the data collection are presented in the following sections.

### 3.1 Data collection

The data related to staff members’ private information were all anonymous. A pseudo staff ID was used by SCAS data controllers to refer to individual staff members. Ethical approval for this research was obtained from the University of Southampton Ethics Committee. The Ethics and Research Governance Online (ERGO) number was 52537.

The original secondary data provided by SCAS included four tables from different data warehouses: (i) stress‐related leave records; (ii) staff information; (iii) incident records, and (iv) work shift records. The data period was from January 1, 2016 to December 31, 2018. In the incident records table, the information recorded stressful incidents attended by each member of staff on each day during the observation period. The work shift table included the work shift that each staff member attended on each day during the observation period. The content included in those tables are presented in Tables 2, 3, 4, 5.

**TABLE 2:** Content of staff information table

Staff information	
Staff ID pseudo	Position title
Gender	Role
Staff group	Latest start date

**TABLE 3:** Content of absence records table

Absence records	
Staff ID pseudo	Absence start date
Absence reason	Absence end date

**TABLE 4:** Content of incidents records table

Incident records	
Staff ID pseudo	Incident number
Incident date	Chief complaint
Abnormal maternity delivery	Child death
Normal maternity delivery	Death confirmed by emergency medical services (EMS)
Infection status type	Mental health diagnosis
Infection status	Safeguarding_Sexual Abuse
Safeguarding form	

**TABLE 5:** Content of work shift table

Work shift	
Staff ID pseudo	Shift duration (hours)
Shift start	

The safeguarding form is a document in either electronic or paper format that ambulance staff must complete if they feel there is a risk to the patient or someone at the scene, who requires a referral to the police, fire, or social services. This type of incident may impose pressure on staff because patients are continuously exposed to some risk that may make them suffer the same incident again. For example, if the patient is abused financially, emotionally, or physically, ambulance staff do not have the authority to stop the incident as they can only provide medical care. In such situations the medical staff can inform the police via a safeguarding form; however, this process can be frustrating and stressful.

### 3.2 Data preprocessing for machine learning and survival modeling

To conduct analysis, the original data were aggregated into a single table by linking their pseudo staff ID. To apply the Cox regression model, all the data must have the same format as the survival data. Namely, there was a column called “duration” to record the actual time when staff were exposed to the stressors before the concerned event happened. The stress‐related leave status column denotes, up to the duration time, whether or not the staff member took stress‐related leave at that point in time.

**FIGURE 1:**
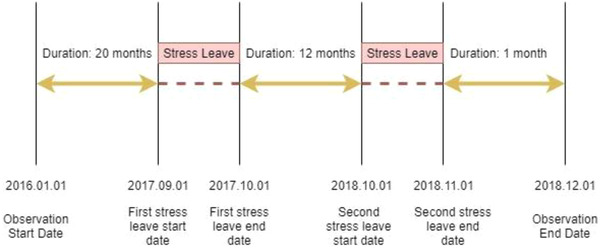
Example of stress‐related leave records

To avoid the information loss from Cox regression, if a staff member has more than one stress‐related leave, the other days of stress‐related leave are considered as new entries into the dataset. It is assumed that a staff member will return refreshed after a stress‐related leave. For example, a staff member that has taken two periods of stress‐related leave within the observation period will have three records in the final dataset. Each record corresponds to the number of incidents a staff member attended between two absences or between the start/end dates of the observation and absences. Figure 1 presents an example of the dataset format. A staff member took two periods of stress‐related leave during the observation period on September 1, 2017 and October 1, 2018, respectively. The duration of each stress‐related leave was 1 month. The first stress‐related leave started 20 months after the start date of the observation. Then the first record contains the number of incidents the person attended within this 20‐month period and, at the same time, the value of duration was 20 months and stress‐related leave status was equal to 1 (1 stands for taking stress‐related leave at the moment; 0 stands for not taking stress‐related leave at the moment). The second record corresponds to the incident that the staff member attended from the end of the first stress‐related leave to the start of the second stress‐related leave, of which the duration was 12 months and the stress‐related leave status was 1. The third record of this staff member was a little different in that it contained the number of incidents they attended from the end of the second stress‐related leave to the end of the observation with the duration of one month.

However, since the staff member did not take any stress‐related leave at the moment of the end of the observation, the stress‐related leave status of the third record was zero. Similarly, if a staff member does not take any stress‐related leave during the observation period, they will only have one record in the dataset over the duration of the whole observation period.

The dataset used by the RF had the same format as that used by the Cox regression, except that there was no “duration” column in the RF since these two models are not able to capture the time of event.

**FIGURE 2:**
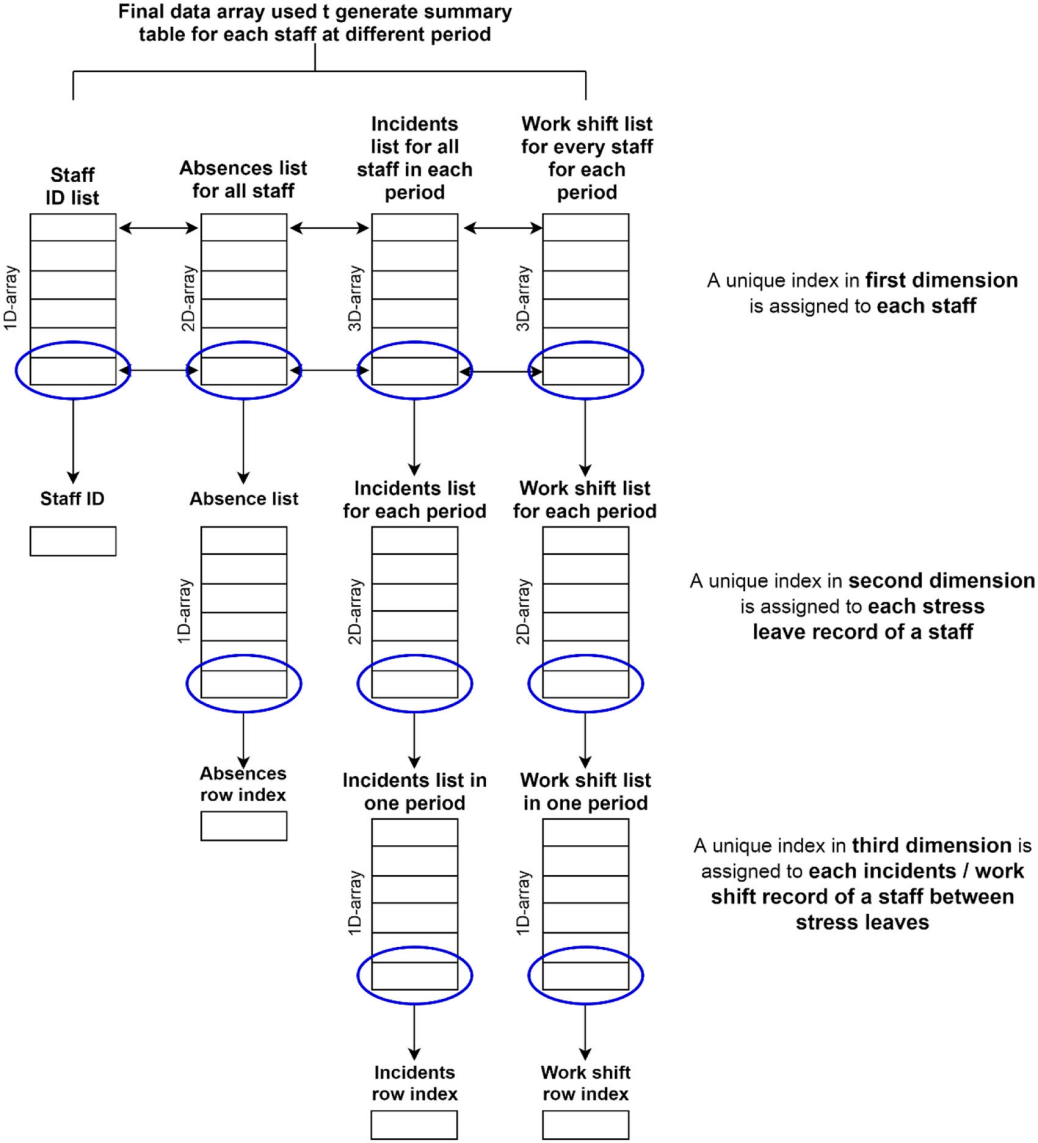
Flow chart of data generation process

Often, secondary data are not recorded in a format suitable for modelling and analysis. The process involves three main steps. *First*, find all the stress‐related leave records, work shift records and incident records of each staff member. *Second*, assign incident records and work shift records to the corresponding working period of each staff member. *Third*, generate the table by accumulating the number of incidents a staff member attended within each working period. In order to manage all the incidents and work shift data within each working period of each individual staff member, the data were organized by using the index position in each dimension of the data array as shown in Figure 2. The process is iterative as it comprises a number of loops. The pseudo‐code for the macro used for extracting the data is presented in Appendix 1.

After data aggregation, the new dataset consisted of 14 independent variables, one extra piece of information for Cox regression, and one dependent variable. Besides the demographic factors, the other factors represented the number of related stressful incidents that an ambulance crew attended within a certain period. For example, if the value of “Child Death” is 5, it means that the staff member has attended five incidents involving child death within a certain period. The data generated are presented in Table 6.

**TABLE 6:** Variables table

Independent variables	Source of variable identification
1. Gender	Experts
2. Position Title	Experts
3. Role	Experts
4. Abnormal maternity delivery	Experts (incident record)
5. Child death	Ross‐Adjie et al. (2007)
6. Death confirmed by EMS	Ross‐Adjie et al. (2007)
7. Infection status	Ross‐Adjie et al. (2007)
8. Mental health diagnosis	Ross‐Adjie et al. (2007)
9. Normal maternity delivery	Experts (incident record)
10. Safeguarding‐sexual abuse	Experts (incident record)
11. Safeguarding form	Experts (incident record)
12. Tenure (months)	Ross‐Adjie et al. (2007)
13. Past stress‐related leave	Roelen et al. (2011), Reis et al. (2011), Wahid et al. (2019), Zaman et al. (2019)
14. Average night shift (1/Days)	Ross‐Adjie et al. (2007)
**Extra information for Cox regression only**	
1. Duration	Roelen et al. (2011)
**Dependent variable**	
1.Stress‐related leave status	Experts

The average night shift was calculated by dividing the total number of night shifts by the total number of days that the medic worked.

To conduct the analysis more efficiently, variables which contain strings were encoded into numerical values. The machine learning algorithms and Cox regression treat these variables as categorical variables. This is required by most of the model algorithms. A summary of the encoded variables and their corresponding numerical values is presented in Table 7.

**TABLE 7:** Summary of encoded variables

Original variable name	Value of variable	Encoded value
Gender	Female	0
	Male	1
	Unknown	0.5
Position Title	Paramedic	0
	NQ paramedic	1
	Clinical mentor	2
	Team leader (B7pay)	3
	Specialist paramedic	4
	HART paramedic	5
	Others	6
Role	Paramedic	0
	Paramedic manager	1
	Paramedic specialist practitioner	2
	Paramedic consultant	3
	Occupational therapist	4
	Others	5

### 3.3 Missing data

Before further analysis, it is important to check the completeness of the dataset. The missing values analysis identified four variables which had missing values: gender, position title, role, and tenure. Their missing percentages were 10.5%, 10.5%, 10.5%, and 14.3%, respectively, see Table 8. White lines indicated the missing values. It was noticed that if one of the values of gender, position title, or role was missing, in most cases, the other two variables’ values were missing too. This may be due to some administrative shortcomings when recording the information. No case had more than four missing values; therefore, all the cases were retained.

**TABLE 8:** Missing values analysis

Variable	Numbers of observations	Numbers of missing values	Percentage of missing values
Gender	1724	202	10.5%
Position Title	1723	203	10.5%
Role	1724	202	10.5%
Tenure	1651	275	14.3%

After getting a general idea of the missing values in the dataset, it was crucial to learn more about the missing values pattern. In this case a Little's missing completely at random (MCAR) test can shed some light on this concern. Since gender, position title, and role were categorical variables, tenure—which was the only quantitative variable—can be tested. The result of Little's MCAR test for tenure gives an expectation maximization mean of 104.2 and a *p* value lower than 0.05. This meant that the missing pattern of tenure was not missing completely at random. Instead, tenure might have some correlation with other variables.

In terms of filling in the missing values, different methods should be wisely chosen according to different situations. For gender, the percentage of females and males was almost the same, but the number of male staff (45%) still slightly exceeded that of female staff (44%). Meanwhile, those staff with unknown gender accounted for 11% of the population. Those with unknown gender were encoded into a new category filling with 0.5.

For role and position title, to avoid the issue of bias on existing groups, missing values were all replaced by “Others.”

### 3.4 Inspection of outliers

During the data processing, we noticed some outliers in the dataset. For example, some of the staff members’ tenures were negative, which was not correct. The reason for this kind of outlier was that these ambulance staff joined the SCAS after the end of the observation period. Another reason was that some staff had taken stress‐related leave before their first start date at work. Moreover, some of the records also showed that some staff members took more than one stress‐related leave in the same time period. These occurrences generated some problems when extracting the data from the data warehouses. All these abnormal cases were deleted to form a final dataset for further analysis with less bias.

## 4 RESULTS

In this section we present descriptive statistics of the participants in this study. We then present results of the classification and Cox regression methods.

### 4.1 Participants’ characteristics

The number of ambulance crew from SCAS involved in this study was 1431. By aggregating the information from various datasets, the number of records in the final dataset was 1892. Among the staff, more than 76% did not take any stress‐related leave from 2016 to 2018; 17% took one day of stress‐related leave; 5% took 2 days of stress‐related leave; and 2% took more than three days of stress‐related leave.

**FIGURE 3:**
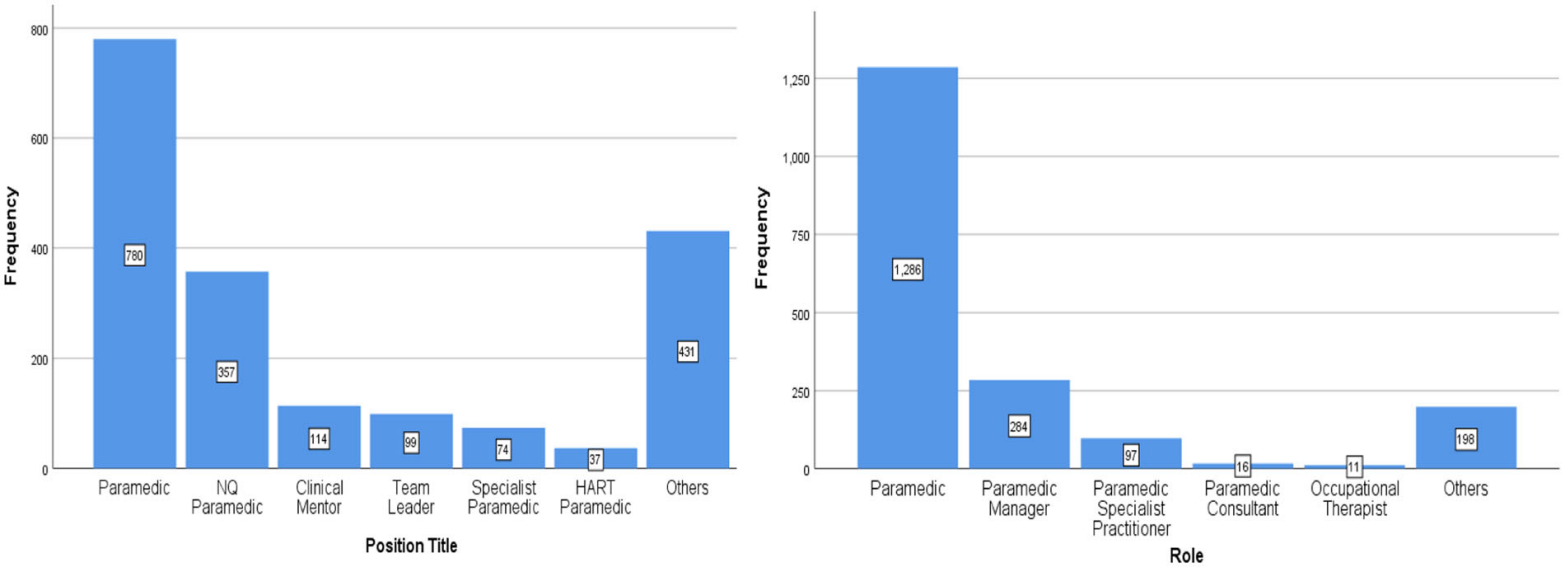
Left: Position title. Right: Role distribution

The position held by the majority of the ambulance crew was *paramedic* but there were still various other roles served in the team (see Figure 3).

### 4.2 Incidents’ profile

**FIGURE 4:**
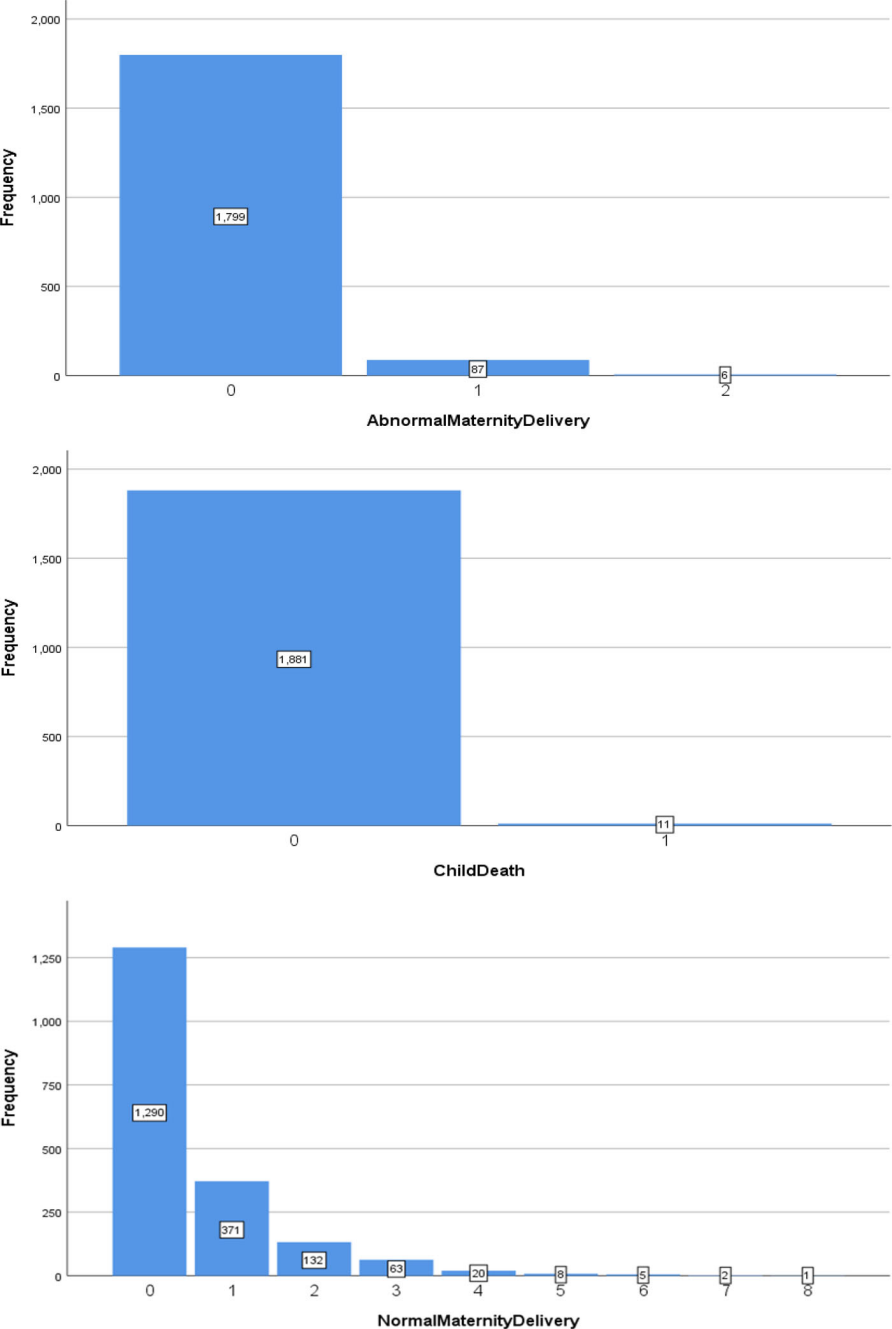
*Top*: The number of AbnormalMaternityDelivery staff has experienced within a working period. *Middle*: The number of Child Death staff has experienced within a working Period. *Bottom*: The number of NormalMaternityDelivery staff has experienced within a working Period

The number of individuals who have not experienced an incident related to abnormal maternity delivery was larger than those who had encountered this situation (see Figure 4 *Top*). This uneven distribution might indirectly denote that the incident rate of this kind of situation was low.

The distribution of child death—see Figure 4 *Middle*—showed the same pattern. Although the occurrence of these stressful events was rare, it was possible that they may have led to extreme pressure. Risk perception studies on stressors among medical staff have identified the events of death and sexual abuse related to a child as two of the top five stressors (Elder et al., 2019). Jewkes (2001) also gave a more explicit insight that 10% of the emergency calls involved children, 5% of which were acutely or critically ill children.

Experiencing this incident once or more than once accounted for 32% among the population. One of the staff members even experienced it eight times within a working period. For the incident related to sexual abuse, it was reassuring to see that the frequency of attending this kind of event zero times within a working period far outweighs the others, but there were still 136 records indicating that a staff member had experienced this at least once within a working period.

**FIGURE 5:**
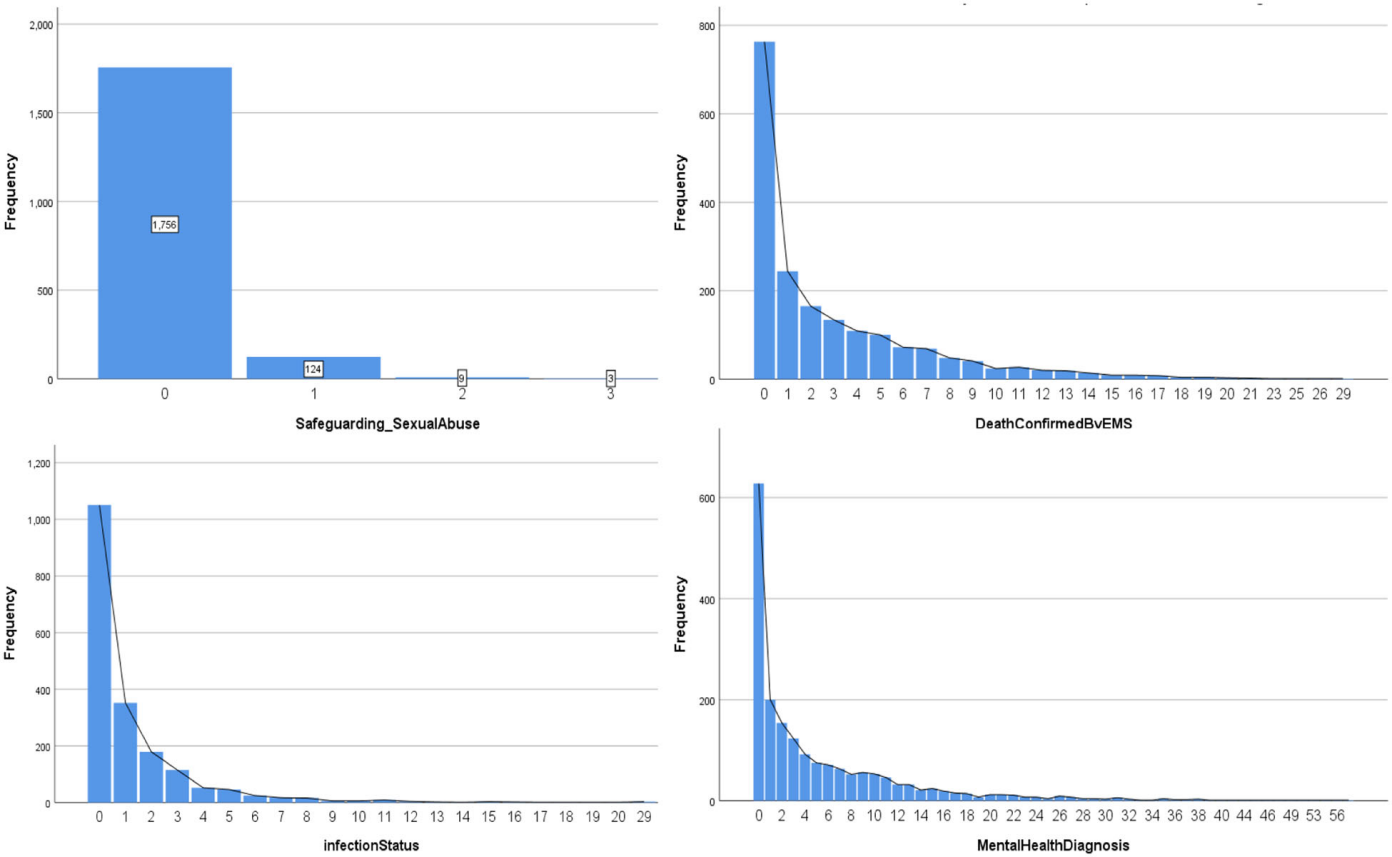
*Top left*: The number of Safeguarding_SexualAbuse staff has experienced within a working period. *Top right*: The number of DeathConfirmedByEMS staff has experienced within a working period. *Bottom left*: The number of infection status staff has experienced within a working period. *Bottom right*: The number of MentalHealthDiagnosis staff has experienced within a working period

Figure 5 also shows that ambulance staff confront death quite often, which implied that the incident rate might be high. Most records showed that staff had experienced at least one incident related to death within each working period. The number of failure incidents that a staff member experienced within a working period ranged from 0 to 29, and the distribution decreased exponentially. Exposure to patients with infectious diseases was another challenge for ambulance staff; however, the incident rate was lower than those of death (see Figure 5). Similarly, it decreased exponentially but with a smaller tail.

Mental health diagnosis referred to the incidents that involved coping with patients who need psychological diagnosis. Ambulance staff encountered this kind of incident the most among all the other incidents in this study. Over 50% of the records revealed that a staff member needed to cope with this situation at least twice within each working period. A small quantity of staff had experienced this more than 20 times within the working period.

### 4.3 Association

To gain more insights into the relationship between independent variables related to staff information and the dependent variable, we applied the Chi‐square test, and the results are presented in Table 9. The associations among gender, position title, role, and stress‐related leave status are described below.

**TABLE 9:** Chi‐square test results

	Chi‐square test	Gender	Position title	Role	Stress‐ related leave status
Gender	Chi‐square		771.38	1892.78	145.45
	*p*‐value		<0.05	<0.05	<0.05
	*df*		12	10	2
Position Title	Chi‐square	771.38		3666.83	67.45
	*p*‐value	<0.05		<0.05	<0.05
	*df*	12		30	6
Role	Chi‐square	1892.78	3666.83		128.30
	*p*‐value	<0.05	<0.05		<0.05
	*df*	10	30		5

The *p*‐values of the Chi‐square test among gender, position title, role, and stress‐related leave status were all lower than 0.05. This implies that we must reject the null hypothesis of which variables were independent from each other. The Chi‐square was extremely large between role and position title, which means that the association may be strong. Therefore, one of them should be left out of the predictive model. In this case, *position title* was not taken into consideration in further analysis.

The correlations among various incidents were not investigated since the correlation among them might be spurious. One of the purposes of this study is identify what kind of incident leads to stress‐related absence. Every kind of incident is different and must be given equal consideration.

### 4.4 Analysis and performance of RF

RF can not only predict whether a staff member will take stress‐related leave but can also shed more light on the relationship between factors and stress‐related leave. To build up an RF model, the first step was to decide the number of decision trees in it. Similarly, the number of trees was determined by conducting several trials. There were 13 independent variables involved in this model.

**FIGURE 6:**
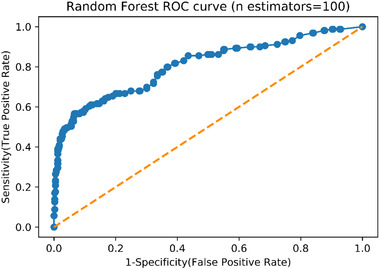
Random forest (100 Estimators) receiver operating characteristics (ROC) curve

This was followed by the comparison of the AUROC of different numbers of trees (see Figure 6). When there were five trees, the AUROC was up to 0.775. When there were more than five trees in a random forest, the differences among the AUROC of different numbers of trees were not that significant although the AUROC was subtly increasing along with the increase in the number of trees. Specifically, the difference of AUROC values for 50, 100, 500, and 1000 trees was 0.003, which is a negligible figure. Therefore, a RF model with 100 decision trees was created for further analysis. Figure 6 presents the ROC curve. The AUROC was 0.806 (95% CI 0.750–0.842) with *p*‐value lower than 0.05. This meant that the value of AUROC was statistically significant, and the discrimination ability of the RF model was good.

RF could depict the relationship between independent variables and stress‐related leave by feature importance using the feature gain. The higher the level of feature importance was, the more important that feature was in helping to classify stress‐related absenteeism in medical responders. Average night shift was the most crucial factor that helped to predict whether a staff member will take a stress‐related leave, followed by tenure, incident related to safeguarding form, incident related to mental health diagnosis, past stress‐related leave, and incident related to death confirmed. These six key features ranked up to 0.8 in total importance. Table 10 presents the level of the feature importance for the RF analysis. The rest of the features only contributed 20% to the classification.

**TABLE 10:** Feature importance of random forest

Variable	Importance	Total importance
Average night shift	0.234	0.234
Tenure	0.174	0.407
Safeguarding form	0.157	0.564
Mental health diagnosis	0.0917	0.656
Past stress‐related leave	0.0794	0.735
Death confirmed by EMS	0.0730	0.808
Role	0.0664	0.875
Infection status	0.0453	0.920
Gender	0.0354	0.955
Normal maternity delivery	0.0297	0.985
Abnormal maternity delivery	0.00703	0.992
Safeguarding_SexualAbuse	0.00662	0.999
Child Death	0.00125	1

**FIGURE 7:**
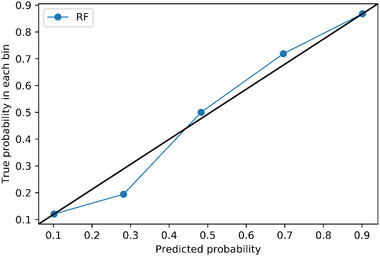
Calibration plot of random forest (*n* estimators = 100)

The calibration plot is presented in Figure 7. The blue line lies close to the black one. This means that, although there were some differences between the predicted probability generated by RF and true probability, the differences were small. However, a Hosmer–Lemeshow test was still needed for a more straightforward measurement. The result showed that the calibration of this RF model was still poor: the χ^2^ = 34.07, the *df* = 8, and the *p*‐value < 0.05.

### 4.5 Analysis and performance of Cox regression

The RF not only revealed prediction outcomes but also revealed how important a feature was when making the classification. However, how the factors affect the stress‐related leave status is still undisclosed. Hence, Cox regression was applied to explore the effect size of the independent variables. Only the six most important factors generated from the RF were considered for the Cox regression model.

#### 4.5.1 Cox regression proportional hazard assumption check

**FIGURE 8:**
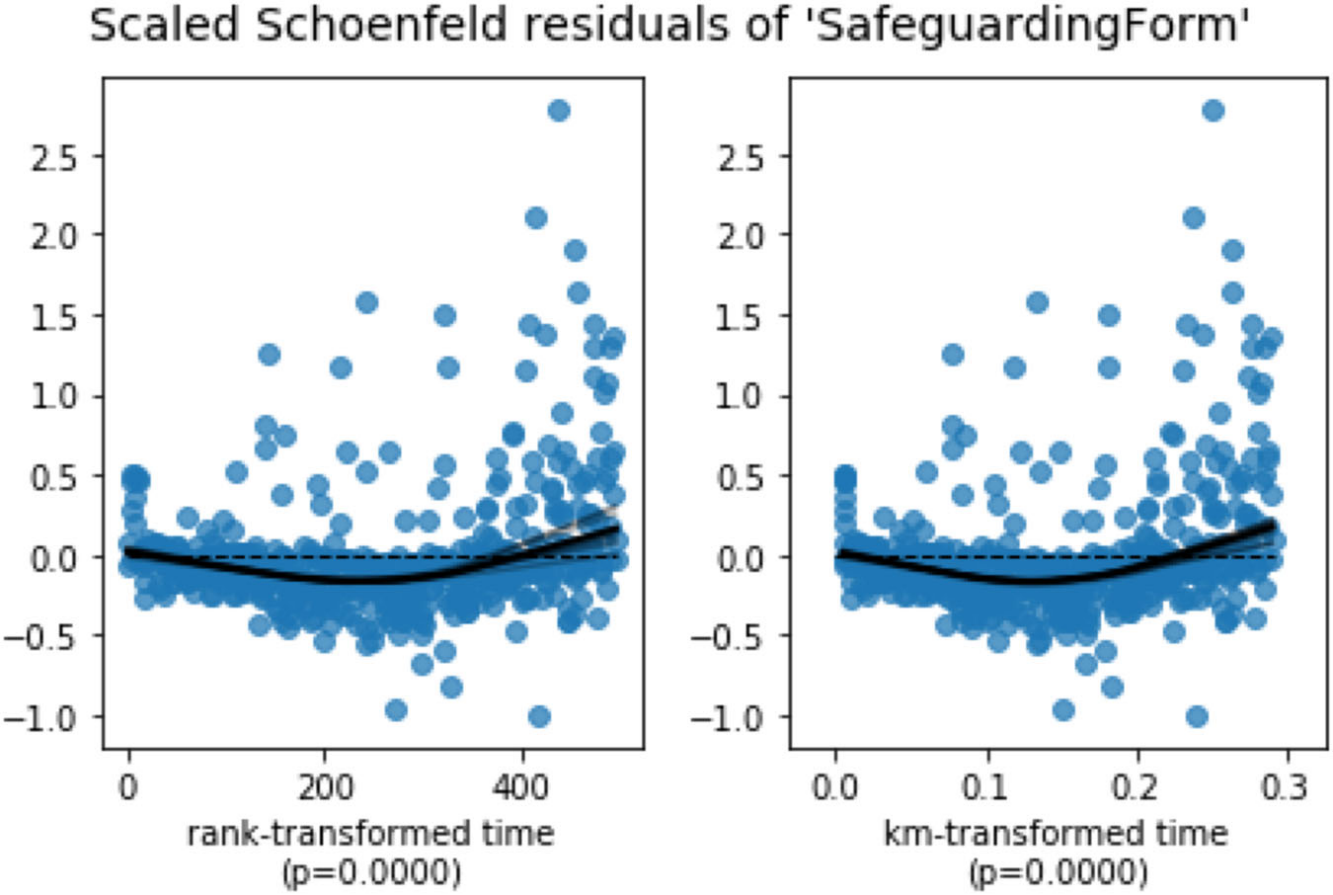
Scaled Schonfeld residuals of “Safeguarding Form”

The scaled Schoenfeld residuals of each variable were plotted (see Figure 8). To pass this test, the Schoenfeld residuals must sum to 0, this indicates that the hazard ratio (or the proportional coefficient) is not changing over time. If the residuals are independent of time, it means that the model does not violate the assumption. The null hypothesis is that hazards are proportional or that the hazard ratio is constant. The 95% confident interval of the residuals must always contain 0, if it does not always contain 0, we must reject the null hypothesis and accept the alternative hypothesis. The residuals are plotted using two transformations of time—*rank* and *KM*. The rank transformation uses the rank of time as the time‐scaling function while the KM transformation uses 1 − Kaplan Meier's product limit estimate as the time‐scaling function. The finding suggested that “Safeguarding Form” failed the proportional hazard assumption test. The statistical assumption check result is shown in Table 11. As can be seen, the *p*‐value of the Safeguarding Form was less than 0.005, which also denotes the violation of the assumption.

**TABLE 11:** Result of proportional hazard test

Variable	Test name	Test statistic	*p*‐value	–log2(p)
Average night shift	Km	0.87	0.35	1.51
	rank	1.72	0.19	2.40
Death confirmed by EMS	Km	3.47	0.06	4.00
	rank	3.36	0.07	3.91
Mental health diagnosis	Km	1.94	0.16	2.61
	rank	1.89	0.17	2.57
Past stress‐related leave	Km	0.06	0.80	0.32
	rank	0.09	0.77	0.38
Safeguarding form	Km	53.06	<0.005	41.49
	rank	48.21	<0.005	37.93
Tenure	Km	0.41	0.52	0.94
	rank	0.42	0.52	0.96

In order to pass the proportional hazard test, we decided to stratify the Safeguarding Form, recognizing that sometimes a transformation of one variable may lead to other variables violating the assumption (Collett, 2003a). The Safeguarding Form was binned into four groups according to its quartile and considered as a stratified variable. The binning of the safeguarding form is presented in Table 12. The value of the Safeguarding Form in the dataset was the number of incidents a staff member attended where a safeguarding form had been completed. The dataset was split into four subsamples based on the stratified variable which was Safeguarding Form, and each subsample has its own baseline hazard but with the identical inference of the coefficients of other variables. Table 13 presents the final variables used in the Cox regression.

**TABLE 12:** Binning of safeguarding form

Binning of Safeguarding Form	
Group 0	*X* = 0
Group 1	0 < *X* < = 2
Group 2	2 < *X* < = 9
Group 3	*X* > 9

**TABLE 13:** Variables in the final cox regression model

*Stratified Variables*:
Safeguarding Form
*Independent Variables*:
Tenure
Average night shift
Average night shift **2
Past stress‐related leave
Past stress‐related leave **2
Past stress‐related leave **3
Death confirmed by EMS **2
Death confirmed by EMS **3
Mental health diagnosis **3

#### 4.5.2 Survival function of stratified variable

**FIGURE 9:**
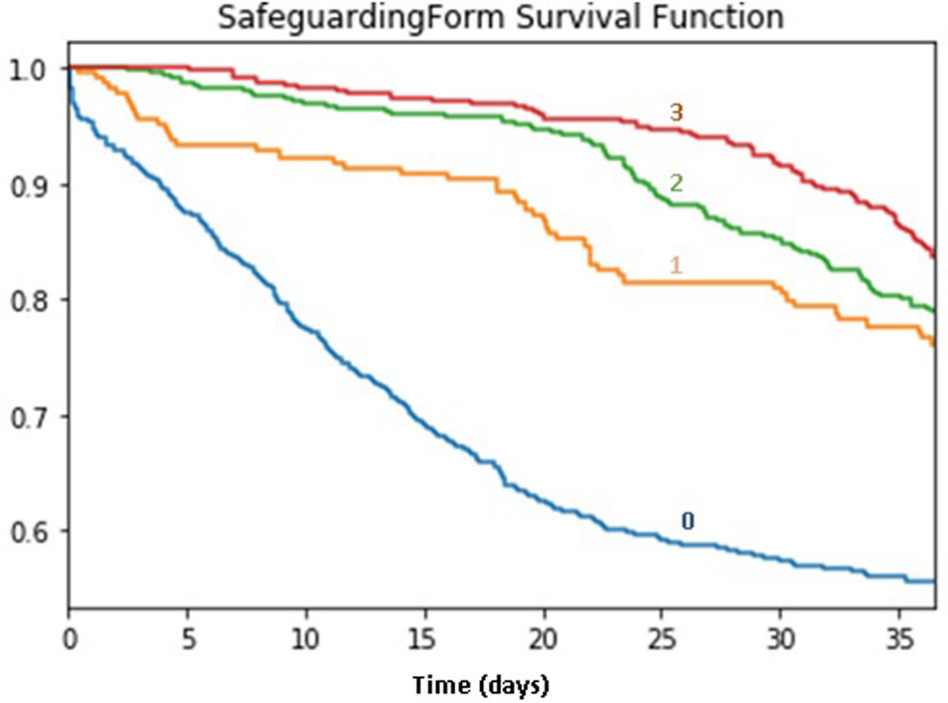
Survival function of safeguarding form

Since Safeguarding Form served as a stratified variable, its effect size related to stress‐related leave could not be included in the Cox regression model. Hence, the survival function of different groups of Safeguarding Form was plotted to gain more information about this variable. Survival function indicated the probability of not taking stress‐related leave over time. A data point represented the probability of not taking a stress‐related leave at that moment. Figure 9 denotes that those who attend incidents that involve filling out a safeguarding form are over nine times less likely to take a stress‐related leave over the whole observation period, followed by those who attended incidents related to filling out a safeguarding form two to nine times. By the end of the observation period, the overall probabilities of different groups of staff not taking a stress‐related leave were all higher than 0.5. The *p*‐value of the log‐rand test was smaller than 0.05, denoting that the survival functions are statistically different.

#### 4.5.3 Coefficients’ interpretation

**FIGURE 10:**
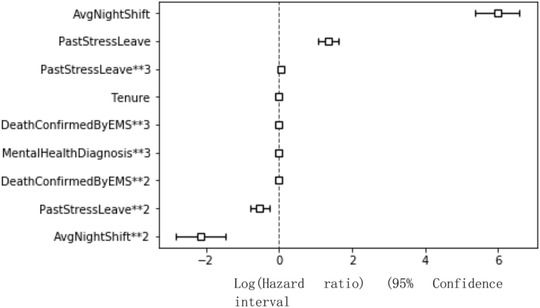
Coefficients plot of cox regression

The sign of a factor's coefficient denoted the relationship between the factor and stress‐related leave. A positive sign of coefficient means that the risk of taking stress‐related leave will increase as the factor increases. A negative sign indicates the opposite situation. All the variables included in the final Cox regression model were significant. Looking at Table 14, the increase of tenure, death confirmed by EMS **3, or mental health diagnosis**3 did not result in increasing the hazard of taking stress‐related leave. However, average night shift and past stress‐related leave record and the square term of death confirmed by EMS affected the hazard of taking stress‐related leave. When there is a one unit increase in the death confirmed by EMS**2, the hazard of taking stress‐related leave will decrease by 7%. In addition, the expected hazard of stress‐related leave in a member of staff whose average night shift is increased by one unit is almost 396 times as high as the baseline hazard when holding all other variables constant. Although the coefficient of average night shift was large, the value of the variable only ranged from 0 to 1, which was significantly smaller than the other variables. Moreover, there was not only a *linear* term of average night shift but also a *square* term of average night shift in the model. A one unit increase in average night shift** 2 results in the expected hazard of taking stress‐related leave being 0.12 times as high as the baseline hazard. The coefficients plot of Cox regression is presented in Figure 10.

**TABLE 14:** Variables’ coefficients of cox regression

	coef	exp(coef)	se(coef)	exp(coef) lower 95%	exp(coef) upper 95%
Tenure	0	1	0	1	1
AvgNightShift	5.98	396.4	0.31	217.02	724.04
PastStressLeave	1.36	3.89	0.14	2.93	5.15
PastStressLeave**2	−0.53	0.59	0.14	0.45	0.77
PastStressLeave**3	0.06	1.06	0.02	1.01	1.11
AvgNightShift**2	−2.14	0.12	0.35	0.06	0.23
DeathConfirmedByEMS**2	−0.02	0.98	0	0.97	0.99
DeathConfirmedByEMS**3	0	1	0	1	1
MentalHealthDiagnosis**3	0	1	0	1	1

#### 4.5.4 Performance of cox regression

Instead of using the ROC Curve, *C*‐index was applied to measure the performance of Cox regression. The *C*‐index of Cox regression was 0.77 and the log‐likelihood ratio test = 558.61 on 9 *df*, –log2(*p*) = 378.03. This means that the predictive ability of Cox regression was good: better than random prediction.

**FIGURE 11:**
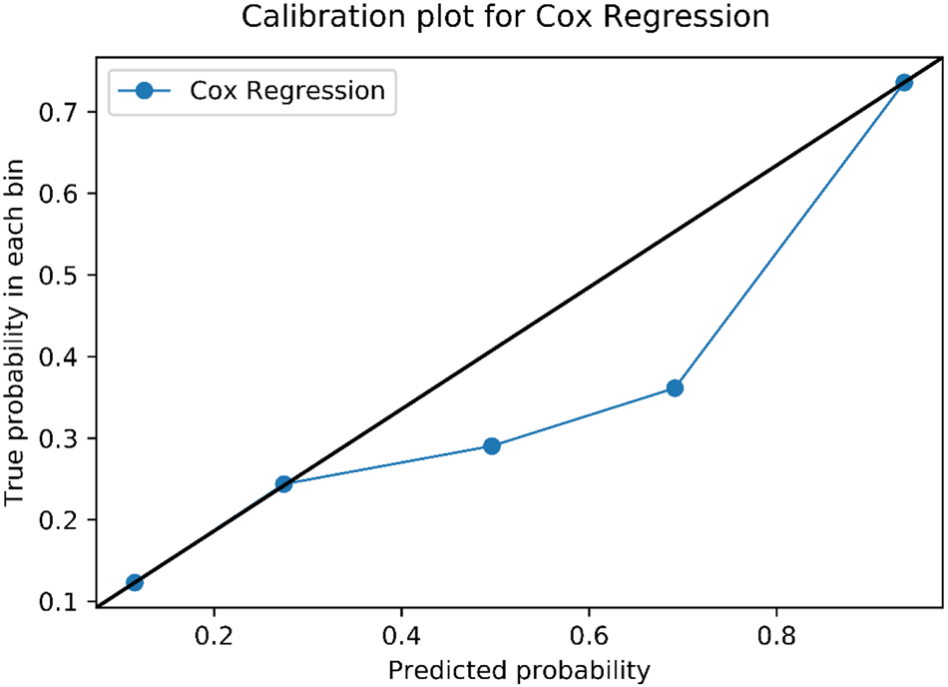
Calibration plot of cox regression

The calibration plot of Cox regression is shown in Figure 11. Three data points perfectly matched on the black line which implies that the predicted probabilities are equal to the true probabilities, but two data points still deviated from the black line. The result of the Hosmer–Lemeshow test was that χ^2^ = 337.30 and *df *= 8, *p *< 0.05. In other words, the Cox regression model may underestimate or overestimate the probability of taking stress‐related leave. The calibration of Cox regression was deficient.

## 5 DISCUSSION AND LIMITATIONS

The findings of this study do not concur with some of the studies in the related industry. Two research papers about occupational stress in emergency departments demonstrated that death or sexual abuse of a child, inability to provide optimum care, violence against staff, heavy workload and poor skill‐mix, mass casualty incident, or environment concerns ranked as the top six stressors (Elder et al., 2019; Ross‐Adjie et al., 2007). Although all four stressors we have identified are included in the top six stressors identified in Elder et al. (2019), the safeguarding form presents counter‐intuitive results. The person who has filled in a safeguarding form a lower number of times has a higher risk of stress‐related absenteeism. This was unexpected. In our view there are three possible explanations. The “healthy worker effect” may have an influence in this result (Roelen et al., 2012). With the “healthy worker effect” the argument is that only “healthy workers” can deal with such incidents and was more likely to be assigned to incidents related to the safeguarding form. Survival bias (Mayeda et al., 2016) is a misleading perception held by those who make decisions and provide support that people who have experienced several traumatic events may need more support than those who have experienced a traumatic event fewer times. Therefore, more resources are put in place to support those who have experienced the traumatic event more often. In this case, people who have completed the safeguarding form more often are more supported than those who have completed the form less often. One other possible explanation is that people who have experienced this traumatic event a higher number of times become more resilient to the event than those who experience the event for the first time. Future research could explore these possible questions further.

At SCAS there is no policy of only sending healthy staff (i.e., with perceived low stress levels) to certain types of jobs. All staff are well trained and are able to deal with the types of incidents that are typically described as stressful. If staff members had self‐reported as being very stressed (or were referred to TRiM–SCAS Trauma Risk Incident Management) they could be individually managed and perhaps allocated alternative (or “light”) duties until their stress levels had lowered sufficiently for them to return to full operational duties. In this research we assume that, after taking stress‐related leave, the staff member is refreshed. This may not be the case for all medical emergency response organizations as this depends on the risk management processes adopted by the organization and, in particular, the perceptions of what are “acceptable” stress levels for a staff member to return to the front line.

The works of Elder et al. (2019) and Ross et al. (2007) are risk perception studies for which nurses have completed a questionnaire to rank the importance of different stressors. While it is important to gather individuals’ perceptions of risks and their synergies, these studies are susceptible to cultural biases (Wildavsky & Dake, 1990). One key difference between risk perception studies and the hard‐data‐driven model that we propose is that the proposed model can quantify the increments in risk of stress‐related absence, which risk perception studies have not been able to do.

It is possible that we have missed some important variables in our model either because they are not recorded or because we do not have access to the relevant digital system. Factors related to stress such as violence against staff (Ross‐Adjie et al., 2007) and frequency of debriefing (Burns & Harm, 1993) are not included in our model; nor is inappropriate coding. A senior staff member in charge of the debriefing session in SCAS mentioned that ambulance staff always felt stress when they attended some incidents with inappropriate coding. Inappropriate coding means that the chief complaint that the ambulance staff received from the record is different from the actual situation at the scene. At the time of this study, SCAS did not record information related to inappropriate coding.

We have collected data from four digital platforms but with the increasing number of digital platforms available, it is possible that we have not included important variables in our model that are recorded in some form of digital system. Two examples of such data is the speed of the ambulance and the traffic intensity. Future research should explore the benefits of including other environment data.

Past stress‐related leave records can show the pattern and trends of stress‐related leave, and can be a reference to future stress‐related leave prediction. Although a few studies focused on predicting stress‐related leave (Nieuwenhuijsen et al., 2006), abundant research predicts general sickness absence (Boot et al., 2017; Notenbomer et al., 2019; Roelen et al., 2011, 2012). In this study, the past stress‐related leave record was one of the strong predictors that helped to determine whether a staff will take a stress‐related leave at a specific time. Similar findings were reported by several studies with respect to sick leave (Navarro et al., 2009; Reis et al., 2011a).

Several studies also mentioned that death or sexual abuse involving a child was extremely stressful but occurred at a very low frequency (Burns & Harm, 1993; Laposa et al., 2003; Ross‐Adjie et al., 2007). Indeed, the low frequency of this kind of incident was also demonstrated in this study—Figure 4 *Middle*—but the relationship between this kind of incident and stress‐related leave is not evident. This may be due to the problem of class imbalance. Fernandez et al. (2017) and Leevy et al. (2018) present methods for dealing with the problem of class imbalance. The synthetic minority oversampling technique (SMOTE) is one of the most popular methods (Nitesh et al., 2002). Future research should attempt to explore different methods to address the problem of class imbalance.

The results of Cox regression showed that, of the six most important factors identified by RF, only four factors—safeguarding form, average night shift, past stress‐related leave, and death confirmed by EMS—had an actual impact on stress‐related leave. Length of tenure and incident related to mental health diagnosis had no impact on stress‐related leave. The ensemble algorithm of RF might be more accurate and convincing. The relationship between stress‐related leave and average night shift, and past stress‐related leave and death confirmed by EMS were not singly linear. The relationship involved square terms and cubic terms of the variables in the hazard function. It was rational that the hazard function was complicated because the probability of stress‐related leave was hard to predict and the mechanism underlying how a factor triggered stress‐related leave was difficult to depict by linear term only.

Another limitation of this study is the lack of internal validation. Due to the nature of the RF that has an inherent bootstrap technique to avoid overfitting, it does not need internal validation. The bootstrapping technique could generate several samples with different data structures by random sampling with replacement. However, Cox regression might need internal validation for modifying the over‐optimism. It is commonly believed that the prediction model will predict more accurately in the subjects used to create the model than in new subjects (Roelen et al., 2012). Therefore, the result of Cox regression might be less reliable and may need further internal validation. The population for this study consists of individuals who work in the same environment, so the results of this study cannot be generalized to the workforce in other industries. External validity should be assessed before applying the results to other working populations.

## 6 CONCLUSION

This study examined the factors that eliciting stress‐related leave and established a model that helps the stress‐related leave prediction. By doing so, SCAS can gain more insight into enduring the provision of appropriate mental support to their ambulance staff, and ensure they are better prepared to deal with the labor loss when some staff really need stress‐related leave. SVC, RF, and Cox regression were applied to address the concerns. There were 13 independent variables in the RF model. These independent variables were selected based on literature and advice from SCAS team members. Only the six most important variables generated from RF were considered in the Cox regression model. Three models were discussed separately based on the following.

In this study, RF identified the six most important factors that significantly contribute to the stress‐related leave prediction. They were average night shift, tenure, the number of incidents related to safeguarding form, the number of incidents related to mental health diagnosis, past stress‐related leave record, and the number of incidents related to death.

Rather than considering only the first stress‐related leave of each staff member, all the stress‐related leave records of each staff member are utilized to establish the Cox regression model, which fits better with the real‐life situation. In terms of measurement of the model performance, both discrimination and calibration are assessed.

The population of this study is homogeneous and limited to the ambulance staff from SCAS. While the results do not necessarily apply to medical emergency response staff from other organizations, the methodology can be applied to any emergency response services—for example, fire fighters, sea rescue, policing, and even the military.

In the immediate future the findings of this research can be used to inform operation risk management. That said, the longer‐term implications can inform law and contractual arrangements, and emergency services’ policies.

## ACKOWLEDGMENTS

We would also like to show our gratitude to colleagues at the South Central Ambulance Service in the United Kingdom for sharing their wisdom with us during the course of this research, we thank the two anonymous reviewers for their insights and the editor for managing this submission.

## PSEUDO‐CODE FOR AMBULANCE RESPONSE DATA FORMATTING

*# read all excel files into tables*

*FOR absence and staff information*:

*Read excel file into table*

*Filter out ‘Staff group’ that is not ‘Allied Health Professionals’*

*Reset index of the table*

*ENDFOR*

*Read incidents excel file into table*

*Read work shift excel file into table*

*Drop rows with negative work duration*

*Reset index of the table*

*Initialize first and last day of observation*

*Remove absences_table, incidents_table and work_shift_table start date earlier then first day or later than last day*

*Reset index of the tables*

*Create a unique staff list by combining staff id from absences_table and staff_info_table*

*# remove duplicated absence*

*Sort absences_table according to ‘Staff ID’ followed by ‘Absence start date’ and ‘Absence end date’*

*FOR second to last row of absences_table*:

*Compare ‘Staff ID’ and ‘Absence start date’ of current row and upper row*

*IF same ‘Staff ID’ and ‘Absence start date’*:

*Drop row with longer duration*

*ENDIF*

*ENDFOR*

*Initialize a 2‐dimensional array to store absence index for each staff id (‘absences_id’)*

*# First dimension corresponds to the staff id at the same index*

*# Second dimension correspond to the absences for each staff id*

*FOR each staff id*

*Find all rows matches with staff id from absences_table*

*Save the row index into an array*

*Save the array into ‘absences_id’ with the row index same as ‘staff_id’*

*ENDFOR*

*Repeat for incidents (‘incidents_id’) and work shift (‘work_shift_id’)*

*Initialize a 3‐dimensional array for incidents between each absence (‘incidents_id_t’)*

*# First dimension corresponds to the staff id at the same index*

*# Second dimension corresponds to the absences for each staff id*

*# Third dimension corresponds to the incidents between two absences*

*FOR each staff id*

*IF no absences*

*Save all incident indices for the staff*

*ELSE*

*FOR each absence of the staff id*

*IF first absence*

*Save all incident indices before first absence*

*ELSE*

*Save all incident indices between current and previous absence found in ‘incidents_id’*

*ENDIF*

*ENDIF*

*ENDFOR*

*ENDFOR*

*Repeat for work shift between each absence (‘work_shift_id_t’)*

*# Generate a table summarising absences and incidents for each staff between each absence*

*FOR each staff id*

*Append each staff info into a row array*

*FOR each period (between two absences, before first absence and after last absence)*

*FOR each incident category*

*Accumulate the incidents between two absences using ‘incidents_id_t’*

*Accumulate total night shift count from ‘work_shift_id_t’*

*ENDFOR*

*Counts duration of the period*

*Calculate average night shift and other parameters*

*Concatenate all information within the period into a row and append to summary table*

*ENDFOR*

*ENDFOR*

Listing 1: Pseudo codes for data generation
